# Supplementary figures and images for: Basal association of a transcription factor favors early gene expression
Source: PLoS Genet. 2025 Jun 16;21(6):e1011710. doi: 10.1371/journal.pgen.1011710 (PMC12187013; doi:10.1371/journal.pgen.1011710)

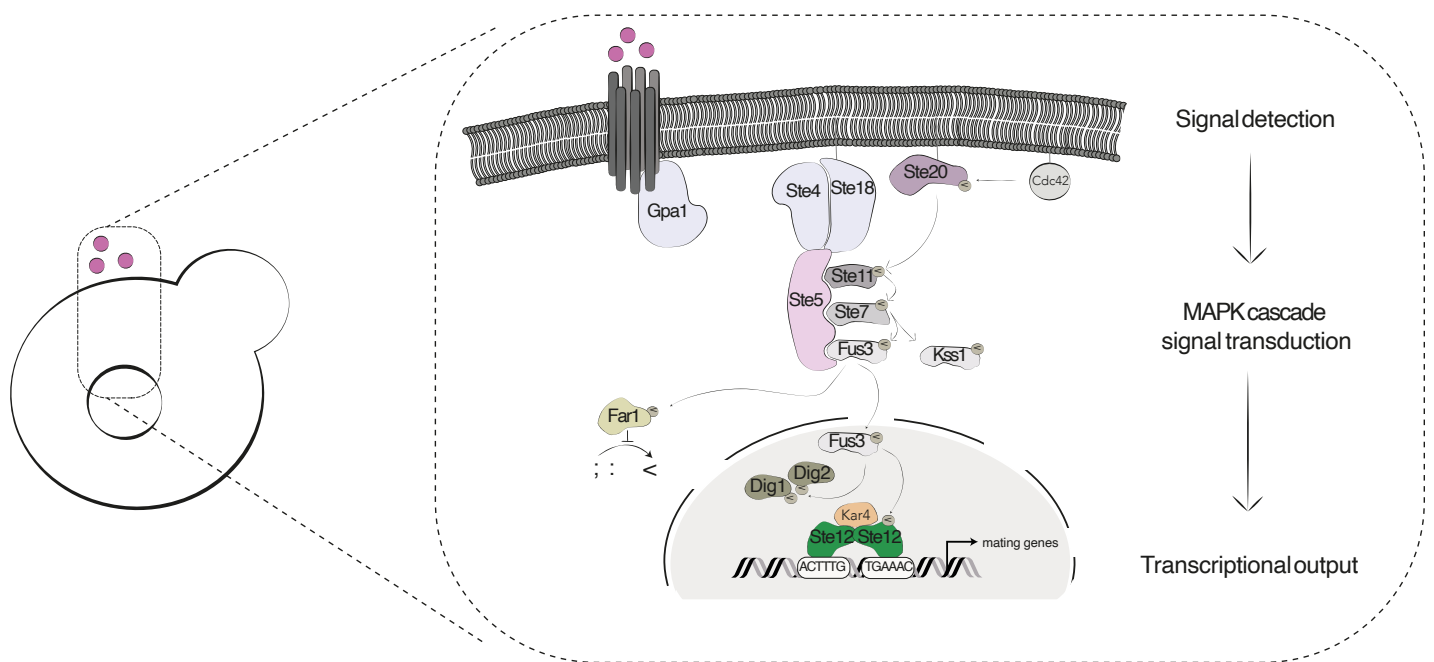

Supplementary Figure 1

Supplement: S1 Fig — The G-protein disassemble and recruits the scaffold Ste5 at the plasma membrane. Then, Ste20 activates the MAP3K Ste11, which phosphorylates the MAP2K Ste7. Ste7 phosphorylates both MAPK Fus3 and Kss1. Fus3 phosphorylates Far1 to arrest the cell cycle in G1. Both Kss1 and Fus3 contribute to the transcriptional response by inhibiting the repression exerted on the TF Ste12 by Dig1 and Dig2. (PDF) [file pgen.1011710.s001.pdf]

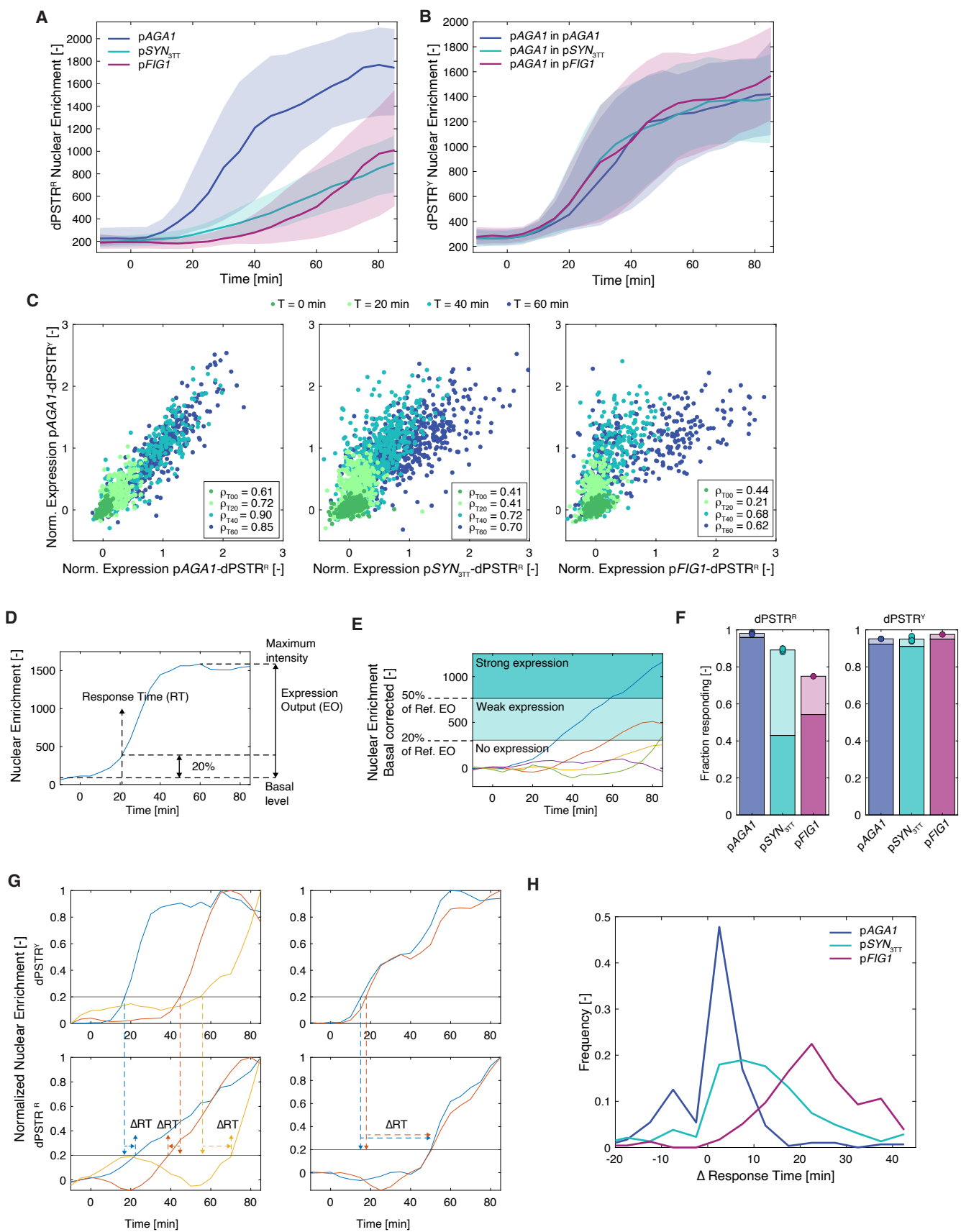

Supplementary Figure 2

Supplement: S2 Fig — A. Dynamics of nuclear enrichment of the dPSTRR under the control of the pAGA1 (dark blue), the pFIG1 (magenta) or the synthetic promoter with two PREs in tail-to-tail orientation with 3 bp spacing (pSYN3TT - cyan). The solid line represents the median of the population, while the shaded area represents the 25- to 75-percentiles of the population. B. Dynamics of nuclear enrichment of the dPSTRY under the control of the endogenous pAGA1 promoter which is present in parallel to the test dPSTRR reporter for the three strains presented in panel A. The response of the pAGA1-dPSTRY serves as a control for the robustness of pheromone induction for all experiments. If the pAGA1-dPSTRY is not induced properly, the experiment will be rejected. C. Correlation of the normalized expression level at 0, 20, 40, 60 min after the stimulus between the pAGA1-dPSTRY (y-axis) and pAGA1 (left), pSYN3TT (middle) and the pFIG1 (right)-dPSTRR (x-axis). The Spearman correlation coefficient for each distribution is indicated in the lower right corner. D. Description of the metrics measured from a single cell trace of nuclear enrichment. The mean of the nuclear enrichment of the first 3 time points is used to quantify the basal level of expression of the trace. The difference between the maximum of the trace and the basal level represents the expression output (EO). When the trace overcomes the threshold set by the 20% of this EO added to the basal level, the response time (RT) is defined. E. To characterize individual single cell traces as not responding, weakly or strongly responding, the mean EO of all the cells of a reference strain is used. In the present case, the reference strain is the pAGA1-dPSTRR construct which is used as a reference. Two criteria are used to define expressing cells. First, the last 5 points of the trace have to be significantly higher than the basal level (sign-test, blue, red, yellow traces). Second, the Expression Ouput of the trace has to overcome the expression [file pgen.1011710.s002.pdf]

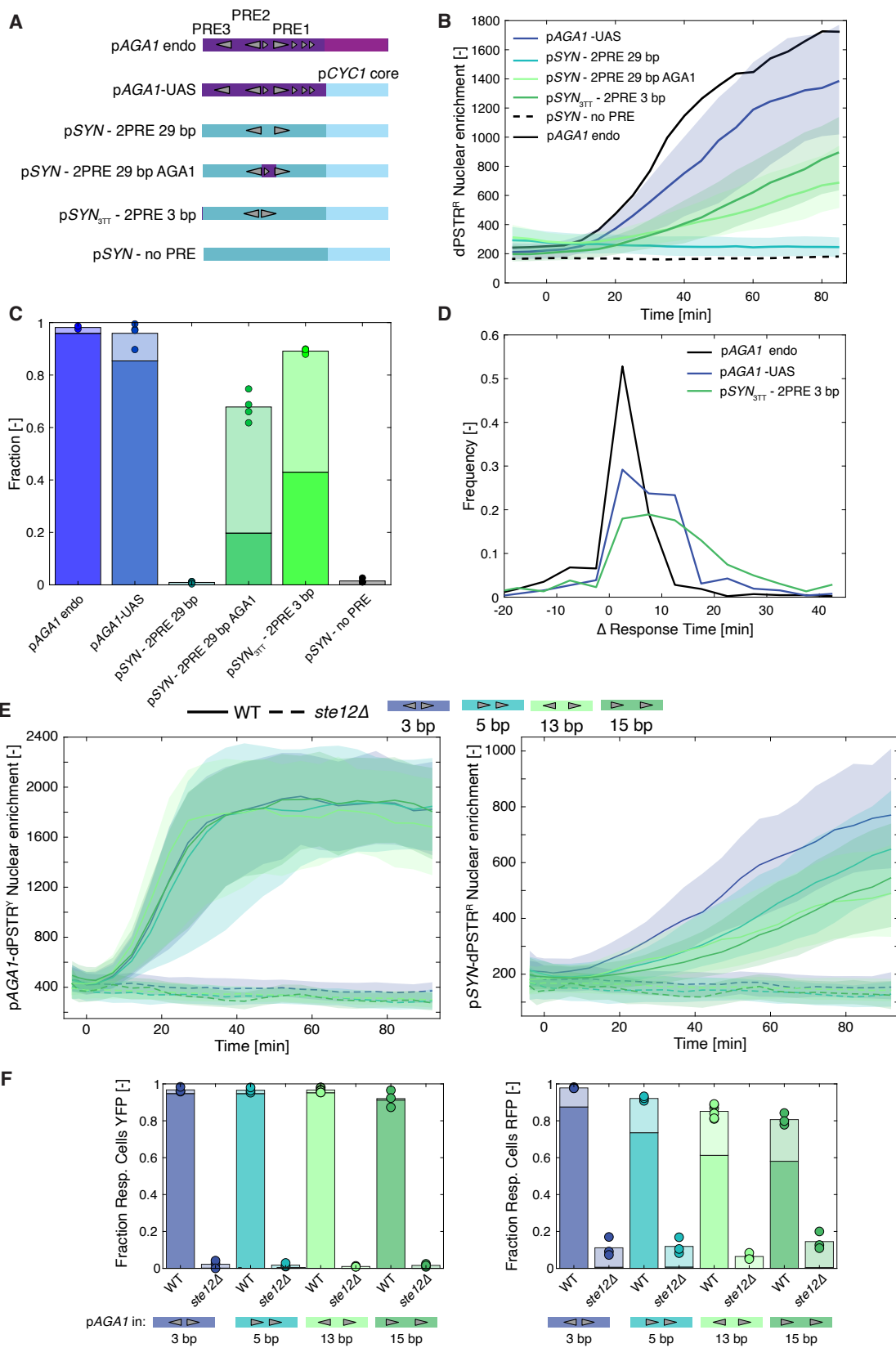

Supplementary Figure 3

Supplement: S3 Fig — A. Scheme of the various promoter configurations tested starting from the pAGA1 endogenous reporter and exchanging the core promoter and testing regulatory regions with a UAS containing various configurations of PREs. B. Dynamics of nuclear enrichment for the dPSTRR under the control of various synthetic promoters. The colored solid lines represent the median of the population and the shaded area, the 25- and 75-percentile of the population. The solid black line is the reference induction from the endogenous promoter pAGA1 and the dashed line is the control promoter without PRE sites. C. Fraction of strongly (dark bar) and weakly (light bar) responding cells relative to the pAGA1-dPSTRR. The total fraction of responding cells from individual replicates is displayed by the markers. D. Histogram of the difference in response time between the tested promoter and the internal reference provided by the pAGA1-dPSTRY. E. Dynamics of nuclear enrichment for the pAGA1-dPSTRY (left) and pSYN-dPSTRR (right) variants in WT (solid line) and ste12∆ (dashed lines) strains. F. Fraction of responding cells in the WT and ste12∆ strains for the pAGA1-dPSTRY (left) and pSYN-dPSTRR (right) variants. (PDF) [file pgen.1011710.s003.pdf]

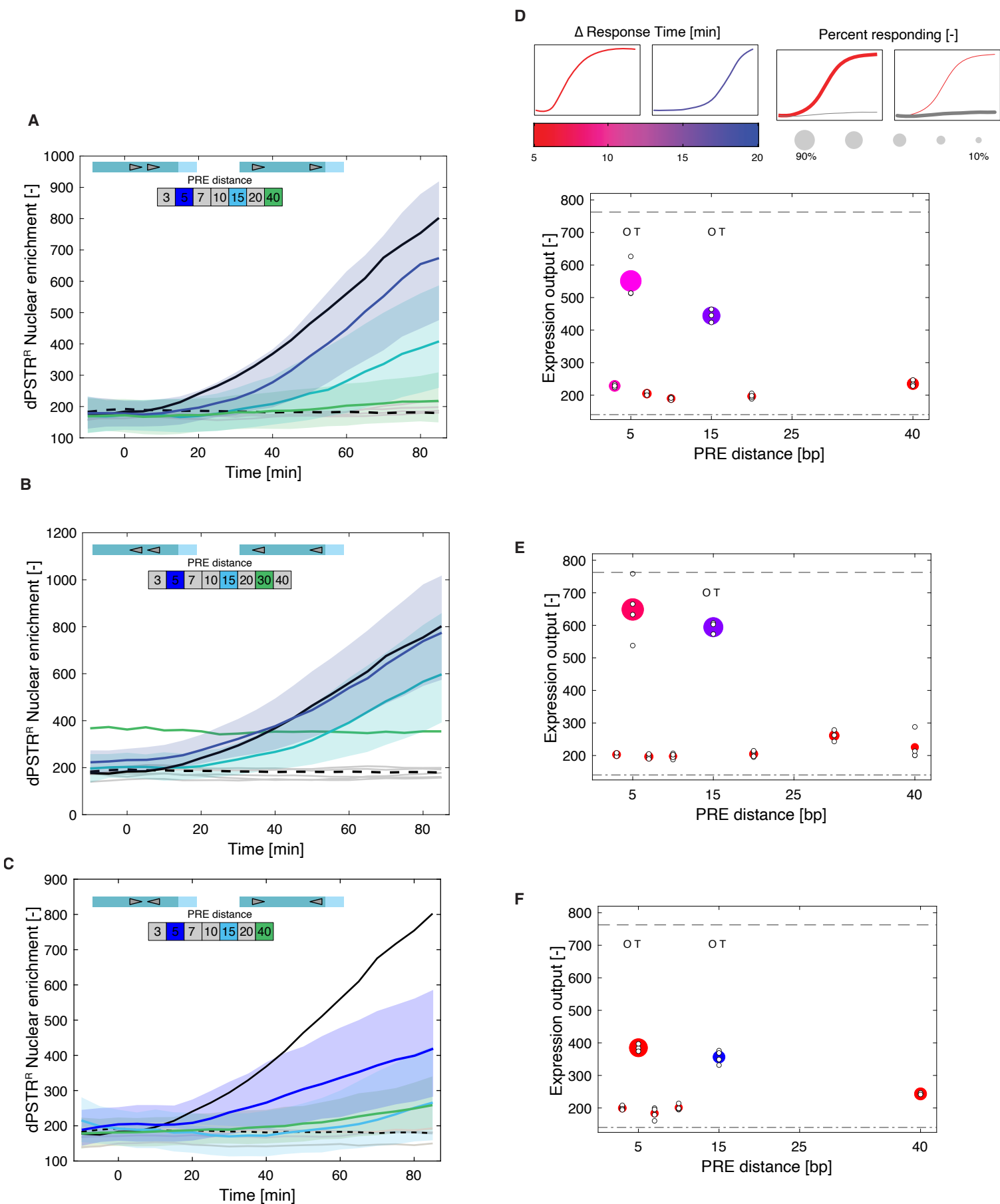

Supplementary Figure 4

Supplement: S4 Fig — A, B and C. Time course of the nuclear enrichment of the dPSTRR for various distances of PRE placed in tail-to-head conformation towards the core (A), in tail-to-head conformation away from the core (B) and in head-to-head conformation (C). Three spacings are plotted in color. The solid lines represent the median and the shaded area the 25- to 75- percentile of the population. Gray lines represent the median of non-functional PRE conformations. The solid black line represents the median of the pSYN3TT reference promoter. The black dashed line is the median of the control synthetic promoter without PRE. D, E and F. Summary graph displaying the expression output, the speed and the fraction of responding cells for various spacings of the two PREs placed in tail-to-head conformation towards the core (D), in tail-to-head conformation away from the core (E) and in head-to-head conformation (F). The color of the marker indicates the difference in response time between the synthetic promoter and the reference pAGA1-dPSTRY. The size of the marker represents the fraction of responding cells. The dashed line represents the expression output and the dashed dotted line the expression threshold calculated based on the pSYN3TT. The O and T indicate a significant difference between the mean of the replicates (t-test: p-val < 0.05) in the timing of induction (T) or in the expression output (O) relative to the pSYN3TT. (PDF) [file pgen.1011710.s004.pdf]

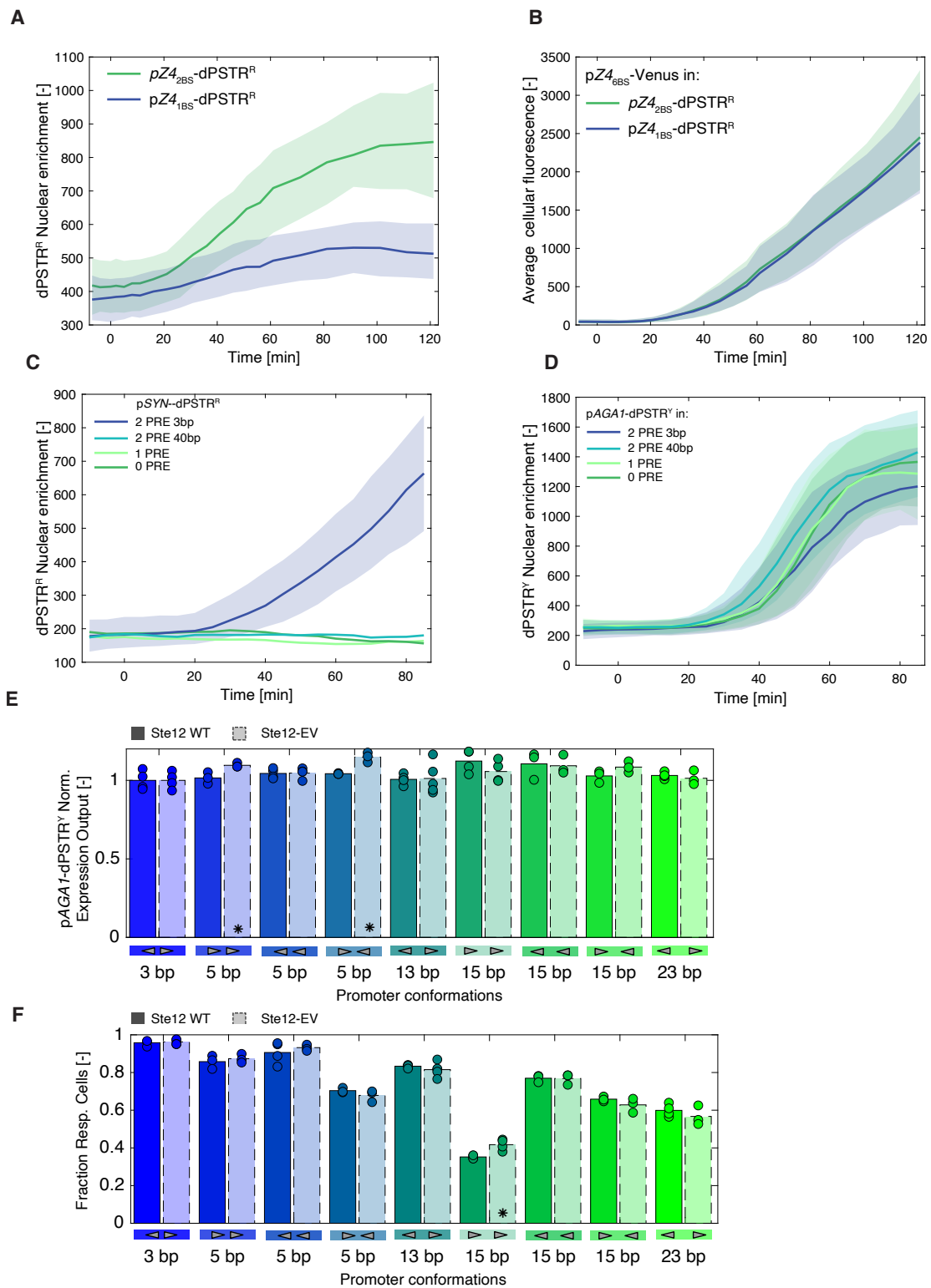

Supplementary Figure 5

Supplement: S5 Fig — A. Dynamics of nuclear enrichment of the dPSTRR under the control of synthetic promoters with one (blue) or two (green) Z4 binding sites (McIsaac NAR 2013) using the synthetic transcription factor Z4-EV upon stimulation with 1µm β-estradiol at time 0. B. Increase in cellular fluorescence as function of time for the reference promoter containing 6 Z4 binding sites and driving the expression of a Venus fluorescent protein which serves as an induction control for the experiment plotted in panel A. C. Dynamics of nuclear enrichment of the dPSTRR by the Ste12-EV upon stimulus by β-estradiol at time 0 under the control of different promoters containing zero (dark green), 1 PRE (light green) or two PREs in tail-to-tail orientation (blue). No detectable nuclear enrichment is observed for the 0 or 1 PRE controls, as well as, the 2 PRE spaced by 40 bp (light blue). If the 2 PREs are spaced by 3 bp (pSYN3TT), the induction is strong (dark blue). D. Dynamics of nuclear enrichment of the control pAGA1-dPSTRY by the Ste12-EV in the strains containing the synthetic promoters displayed in panel C.E. Comparison of the inducibility of pAGA1-dPSTRY with the Ste12 WT (solid borders) or the Ste12-EV (dashed borders). The Expression Outputs for the pAGA1 reporters induced by the Ste12 WT or the Ste12-EV were normalized relative to the Expression Output of the reference pSYN3TT sample. The bar represents the mean response of the replicates shown by the circles. A significant difference between the normalized EO Ste12-WT and Ste12-EV is indicated by a star (t-test: p-val < 0.05) F. Fraction of responding cells for the pSYN-dPSTRR variants with the Ste12 WT (solid borders) or the Ste12-EV (dashed borders). The bar represents the mean response of the replicates shown by the circles. A significant difference between the fraction of responding cells between Ste12-WT and Ste12-EV is indicated by a star (t-test: p-val < 0.05). (PDF) [file pgen.1011710.s005.pdf]

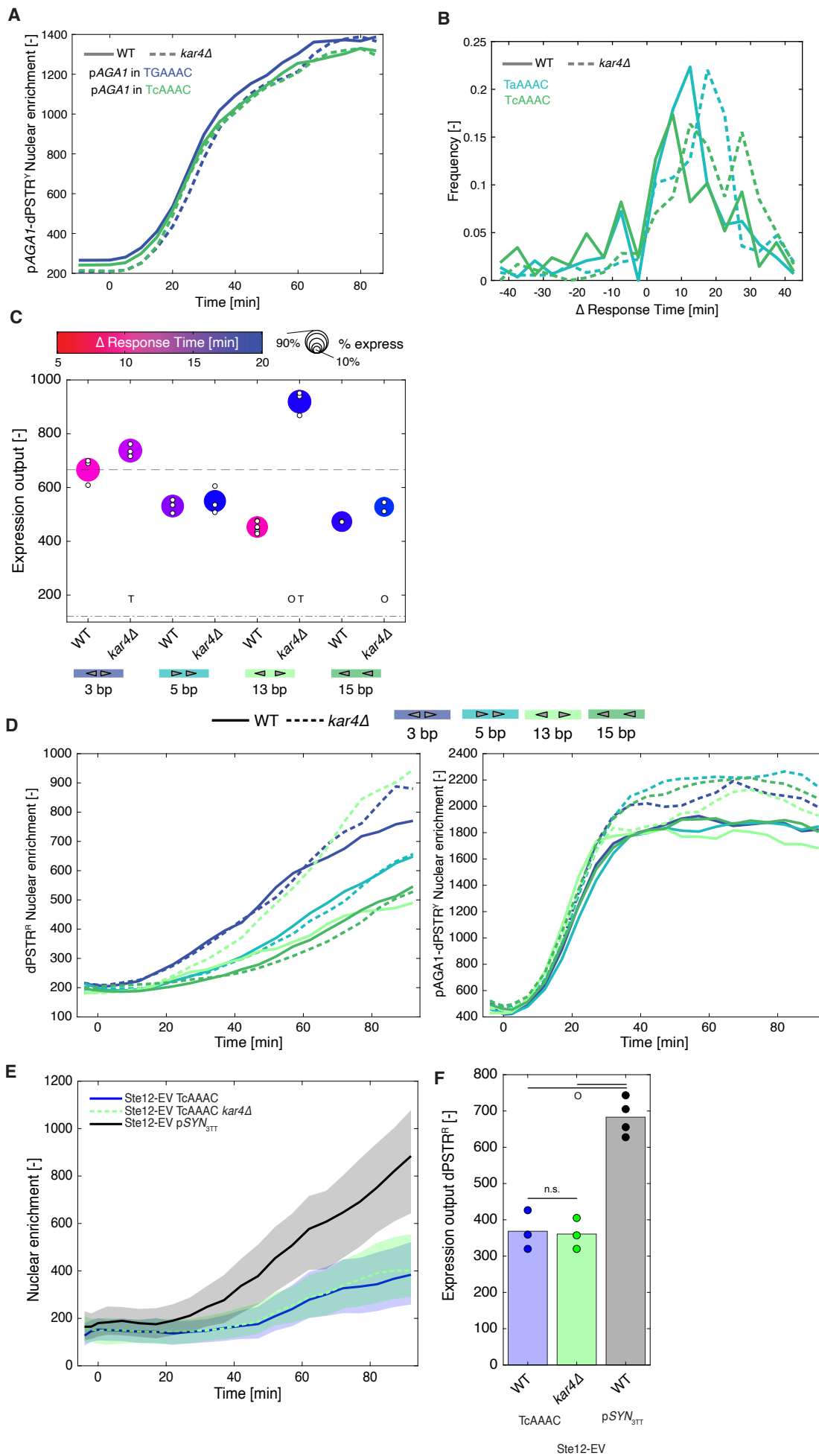

Supplementary Figure 6

Supplement: S6 Fig — A. Dynamics of nuclear enrichment of the pAGA1-dSPTR-Y in WT (solid lines) and kar4∆ cells (dashed lines). B. Histograms of the difference in response time between the tested promoter and the internal pAGA1-dPSTRY reference for WT (solid lines) and kar4∆ cells (dashed lines) for two different non-consensus PRE sequences associated to one consensus PRE. C. Summary graph displaying the expression output, the speed and the fraction of responding cells for promoters with various PRE conformations in WT and kar4∆ cells. The color of the marker indicates the difference in response time between the synthetic promoter and the reference pAGA1-dPSTRY. The size of the marker represents the fraction of responding cells. The expression output of individual replicates is indicated by small white dots. The dashed line represents the expression output and the dashed dotted line the expression threshold calculated based on the pSYN3TT in WT cells. The O and T indicate a significant difference between the mean of the replicates (t-test: p-val < 0.05) in the timing of induction (T) or in the expression output (O) between the WT and kar4∆ strains for the same promoter. D. Dynamics of nuclear enrichment of the pSYN-dPSTRR variants (right panel) and pAGA1-dSPTR-Y (left panel) in WT (solid lines) and kar4∆ cells (dashed lines). E. Dynamics of nuclear enrichment of the pSYN-dPSTRR with two PRE spaced by 3 bp in tail to tail orientation with one mutated PRE (TcAAAC) in WT (solid lines) and kar4∆ cells (dashed line) with the chimeric Ste12-EV promoter and stimulated with β-estradiol at time 0. F. Expression output of the strains measured in panel C. The O indicates that the pSYN3TT expresses significantly stronger than the two strain with the mutated PRE, which both express to the same level. (PDF) [file pgen.1011710.s006.pdf]

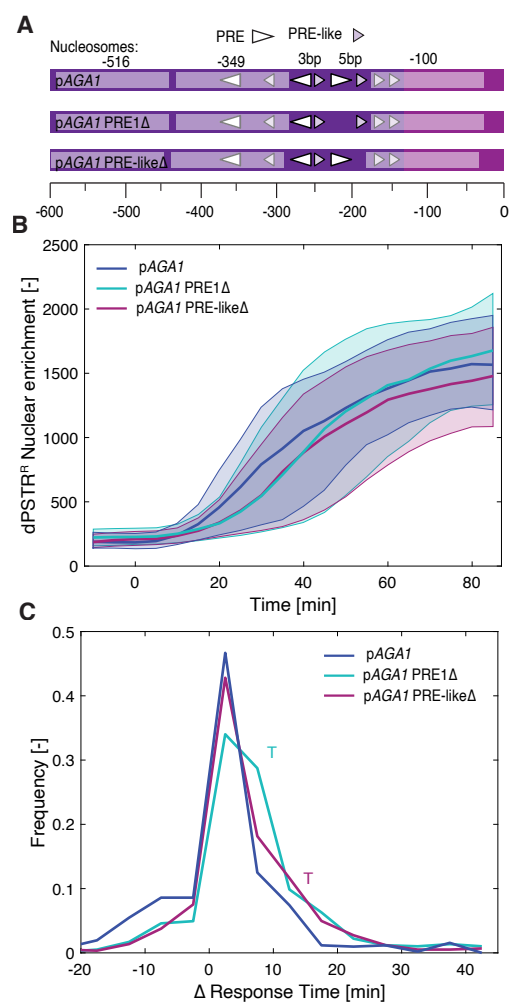

Supplementary Figure 7

Supplement: S7 Fig — A. Scheme of the pAGA1 endogenous promoter, which contains three consensus PRE sites and at least five non-consensus ones. PRE2 together with a non-consensus PRE spaced by 3 bp in tail-to-tail orientation are essential for the inducibility of the promoter. PRE1 (closest to the core) is spaced by 5 bp from a non-consensus site in tail to head conformation. PRE1 or its associated PRE-like have been mutated. B. Dynamics of nuclear enrichment of the dPSTRR under the control of the endogenous (dark blue) or the mutated (light blue or magenta) AGA1 promoter. The solid line represents the median of the population and the shaded area the 25–75- percentile of the population. C. Histogram of the difference in response time between the tested promoters and the internal pAGA1-dPSTRY reference. The T indicates that the histograms for the two mutated promoters are significantly different from the endogenous promoter using a Wilcoxon rank sum test. (PDF) [file pgen.1011710.s007.pdf]
