# Supplementary material for: Basal association of a transcription factor favors early gene expression
Source: PLoS Genet. 2025 Jun 16;21(6):e1011710. doi: 10.1371/journal.pgen.1011710 (PMC12187013; doi:10.1371/journal.pgen.1011710)
Supplement: Attachment — Submitted filename: ResponseReviewers.pdf [file pgen.1011710.s010.pdf]

Reviewer #1

In this manuscript, the authors examine kinetic response of a series of artificial reporter genes regulated by the mating pheromone responsive transcription factor Ste12 in yeast. For these studies they used a dynamic Protein Synthesis Translocation Reporter (dPSTR) which enables reliable measurement of gene expression using fluorescent reporter proteins. The veracity of this system was initially verified using the pheromone responsive AGA1 promoter. They then examine response of a variety of synthetic promoter constructs using the CYC1 core promoter element where they examine variations in organization of pheromone response elements (PREs) at upstream locations. From their studies they conclude that initial rapid induction of pheromone responsive promoters is encouraged by basal association of a Ste12 dimer in a nucleosome depleted upstream promoter region.

Overall, this is an interesting study which examines the effect of Ste12 binding site positioning on kinetics of gene expression in response to pheromone. Ste12 is regulated by MAPK signaling in yeast, and represents an important model transcription factor for growth factor regulated gene expression in eukaryotes. The system designed to examine effects of PRE spacing and orientation for kinetic analysis of expression is quite elegant and the results are mostly clearly described. A similar study detailing effects of PRE positioning for pheromone responsive expression of artificial promoters was previously described, but only using single time point measurements (Ref. 25), This report extends upon these observations using more detailed kinetic analysis. However, a limitation of this study is that the central conclusions are speculative in that the authors have not conclusively demonstrated that rapid induction of pheromone responsive promoters are mediated by a Ste12 protein dimer or within a nucleosome depleted region. Furthermore, I am not sure how relevant the results are for explaining overall pheromone response, considering that Ste12 interacts with multiple additional DNA binding factors, including at least MCM1, a1, alpha2, and likely others. This is not discussed anywhere in the manuscript. Also, all of the conclusions are based on reporter gene expression assays, and there are no results presented to quantify interaction of Ste12 with the artificial promoters, or verify nucleosome occupancy. Consequently the major conclusions are based on vastly over interpreted observations.

We thank the reviewer for his positive assessment of our work. We agree with the reviewer that Ste12 regulation on endogenous promoter is complicated by the fact that Ste12 can interact with additional players, however our objective with this study was to simplify this problem and focus on Ste12 which is responsible for a large fraction of mating genes induction. Mcm1, a1 and alpha2 are important for small subset of cell-type specific genes. We have included this additional element in the introduction of the manuscript.

In the revised version of the paper, we have have performed additional experiments and included biochemical measurements to validate some of the observation made with the live-cell microscopy experiments and to confirm the importance of nucleosome depletion and Ste12 basal association for rapid gene induction.

Specific comments:

- The effect of PRE conformation for induction of pheromone responsive genes is complicated and could even be affected by which core promoter element is used, this is not examined in the present study (this is just a comment, not a suggestion for additional data). As mentioned above, response to pheromone involves interaction of Ste12 with multiple additional proteins, some of which have likely not been identified. This should be clarified in the text.

In Supplementary Figure 3, where the engineering of the synthetic reporter is detailed, we see a clear effect of the core promoter on the level and speed of gene

induction. The p*AGA1* core results in faster and higher expression compared to the selected p*CYC1*. We have stressed this fact in the result and discussion sections of the paper. In addition, we have also tested the inducibility of our various p*SYN* in strains where *STE12* has been deleted and verified the strict dependence of our synthetic promoters on the presence of this transcription factor (Supplementary Figure 3E and F).

- Relating to the above comment, in Figure 2A, the authors show schematics of Ste12 protein bound to 2 PREs. Are these illustrations based on structural analysis or simply speculation? This should be clarified.

We have slightly modified the scheme in Figure 2 to illustrate more precisely our improved results obtained with the Ste12-EV hybrid TF. These drawings represent the fact that Ste12 consists of a well-structured DNA binding domain and a flexible C-terminus containing the activation domain. We have changed the legend of the figure and the text to make this more explicit.

- The results examining the effect of Kar4 are confusing. First of all, Kar4 is thought to bind to its own cis-element on a subset of pheromone response genes, this is not discussed in the text. Furthermore, the observation that Kar4 contributes to rapid activation of the artificial promoters is contrary to its known role for induction of delayed genes during pheromone response. Without some mechanistic information regarding interaction of Ste12 and Kar4 protein, the significance of these results are questionable.

SGD describes KAR4 as a mating transcription factor and early results from the Rose lab suggest that Kar4 binds DNA and these researchers identified a putative bind sequence [Lahav Molecular and Cellular Biology, 2007]. We think that these data are not completely convincing and we priviledge a model where Kar4 contributes to mating gene induction via its binding to Ste12. Multiple evidences align with this hypothesis:

- Newer data from the Rose lab analyzing point mutants of Kar4 defficient for mating suggest that Kar4 acts via an interaction with Ste12 (Park et al. PLOS Genetics, 2023).
- The yeast epigenome project (yeastepigenome.org) has identified a binding for Kar4 on 75 sites in the genome which corresponds to two Ste12 binding sites in head to tail orientation spaced by 5 bp.
- AlphaFold 3 does not predict an association of Kar4 to the DNA, but interestingly, it can predict an interaction between a Ste12 dimer bound to DNA and Kar4.
- In a previous study, we have shown by Ch-IP that Kar4 associates with promoters of both late and early genes (Aymoz MSB 2018).
- In agreement with the Rose lab data, in this same study, we have observed a strong influence of the deletion of *KAR4* on the late genes, However, our dynamic measurements also revealed a mild effect of the deletion of *KAR4* on the induction of intermediate genes.

Taken together, these evidences rather suggest that Kar4 is present wherever Ste12 is bound and speaks against a specific association of Kar4 to the promoters of the late genes.

We don't think that the data presented in this new study are in contradiction with the known role of Kar4 for the induction of late genes. Our data favors a model where Kar4 is associated with the Ste12 dimers on the promoter of all mating genes to stabilize the TF-DNA interaction when necessary:

- On early mating genes like p*AGA1* and our ideal p*SYN*$_{3TT}$, the stabilizing effect of Kar4 is limited because the DNA sequence offers already a strong binding platform for Ste12.
- In less favorable promoters, such as the ones where one of the 2 PRE is mutated (Figure 3), Kar4 starts to exert its stabilizing function. Therefore, we see a delay in induction in *kar4Δ* cells for these promoters.
- For late genes, in absence of Kar4, the binding of Ste12 is weakened to an extent where the induction of these genes is either absent or severely delayed.

In the revised manuscript, we have attempted to gain a bit more mechanistic insights by deleting *KAR4* in additional strains. While we observe only a limited effect of the absence of Kar4 of the 5 and 15bp head to tail conformation, we see a strong effect both on the level and dynamics of induction for the pSYN$_{13TT}$. In a strain, using the chimeric Ste12-EV TF, with a weakened PRE TcAAAC, we observe an identical expression output in presence or absence of *KAR4*. These data suggest, that the effect of Kar4 is pronounced when the PRE are in tail to tail position and that the effect on the expression output is conferred by an interaction between Kar4 and the activation domain of Ste12.

We have included a new section in the discussion to interpret these results

- The conclusions are based on the presumed positioning of nucleosomes and binding of Ste12, this would be strengthened considerably with biochemical analysis using ChIP-PCR, for any of the experiments described, including those involving insertion of the putative nucleosome displacing sequences in the synthetic promoter. This would be important to support statements as follows "when the nucleosome is bound, no transcription takes place. In some cells, the histones can be displaced by the binding of Ste12, which remains stably associated to the promoter to induce a sizable but delayed expression".

Following the reviewer suggestions, we have performed Ch-IP of Ste12 on some of the modified *AGA1* promoter variants and used MNase assays to monitor the eviction of the nucleosomes at this same location. These new data which largely confirm the conclusions drawn from our live-cell experiments are presented in Figure 6.

Minor comments:

- The authors need to use proper genetic annotation throughout the manuscript, genes should be noted in italics.

We have modified the manuscript to adhere to this nomenclature.

- *Page 8, the phrase "slow mating promoters", should probably read something like delayed pheromone responsive promoters.*

The wording of this sentence has been revised

Reviewer #2

This work analyzes how promoter architecture dictates the pattern of transcriptional induction. The system being studied is the transcriptional response to yeast mating pheromone, which involves a transcription factor Ste12 and its DNA binding sites known as PREs (pheromone response elements). To help shed light on what determines why different transcripts are induced with different kinetics, magnitudes, and/or population heterogeneities, the authors take a systematic approach involving the construction of synthetic reporter constructs. These synthetic reporters allow the authors to dissect the key determinants by varying individual promoter features, including the number of PREs as well as their spacing, orientation, sequence optimization, distance to transcriptional start site, and positioning relative to nucleosome-occupied regions. The results convincingly show that each of these parameters makes important contributions to the transcriptional response behavior.

Overall, the experiments are sound and the data are of high quality. A primary strength of the study is that the systematic approach allows the individual features of promoter architecture to be isolated from the others, which provides confidence that the change in expression pattern can be properly attributed to the feature being varied. The main weakness of the manuscript is that there are several places where the authors do not appropriately distinguish between observations and interpretations; that is, they make statements about parameters that they did not actually measure, and hence the claims do not seem justified. This leads to some reservations about whether the findings are overinterpreted.

We thank the reviewer for his positive comments and we hope that the additional measurements performed and the rewording of the text answer his concerns.

Below are a variety of issues that I think would be valuable for the authors to elaborate upon, clarify, or reinterpret.

1. There are several places where findings and interpretation should be disentangled:
(a.) On page 8, a Section Heading states "Kar4 stabilizes Ste12 binding to the promoter". In fact, there are no measurements of Ste12 binding, and no measurements of Kar4 stabilization of this binding. The text should more accurately state and emphasize what is demonstrated, and then describe how the findings might be interpreted.

We agree with the reviewer and we have revised this section which includes additional measurements performed in *kar4Δ* cells. We have added a section in the discussion to separate our observations from the interpretation of the results.

(b.) In the same section on pg 8 and the relevant Figure 3, there are six PRE variants with deviations from the consensus sequence. These are described as having different Ste12 binding affinities (e.g., Fig 3 legend title: "low affinity PRE sites"). But the authors do not explicitly state if the Ste12 binding affinities for these specific sequences has been previously measured, and, if so, whether the rank order of binding strength fits with the progressive changes in transcriptional magnitude observed. Consequently, it is unclear if the interpretation of these experiments is based primarily on supposition or on established evidence for changes in affinity.

The affinity of Ste12 for some of these sequences has been tested *in vitro* by Su et al. (FEBS Letters 2010) using competition assays. Relative to the consensus sequence TGAAAC set at 1.0, TaAAAC (0.81) TGAgAC (0.02) and TcAAAC (0.03) showed lower affinities towards Ste12. We do not observe such drastic differences in the induction of the reporter between these mutated PRE sites. However, in our promoter, the mutated PRE is associated with a consensus PRE, which likely contributes to the stabilization of the Ste12 dimer on the promoter. This dual PRE

configuration has been tested *in vitro* by Dorrity et al. (PNAS 2018) and has shown that only limited variations were allowed in the consensus sequences within the TGAAAC motif. The ranking of the data in Figure 3 is based on the expression output and the fraction of responding cells that we quantified. Unfortunately, to our knowledge, existing dataset cannot explain the large difference that we observe between a TGcgAC versus TGggAC. We have modified the text to provide a more detailed explanation of these data.

(c.) Pg 11, top: "...these results demonstrate that nucleosomes prevent the basal association of Ste12 outside of the NDR". The claim of what the results "demonstrate" seems like an overinterpretation. This study contains no measurements of basal association or nucleosomal occupancy. The text should more accurately describe what was measured and observed, and then describe the basis for the eventual interpretation (and the basis for confidence in its likelihood). Even if the interpretation is reasonable, it seems inappropriate to muddy the distinction.

We have performed biochemical experiments to quantify the binding of Ste12 and test the eviction of nucleosomes on the *AGA1* promoter to verify our conclusions. In addition, we have tried to improve the manuscript by separating more clearly the description of the results and their interpretation.

2. Page 7, middle: "Additionally, Ste12 controls its own expression, thus the level of the chimeric transcription factor is probably lower than the ones of the endogenous protein in absence of beta-estradiol." The logic here is not compelling. Just because Ste12 increases its own expression, there is no inherent basis for expecting that the eventual levels are greater rather than lower than the beta-estradiol induced levels. For example native STE12 transcripts might increase from 0.1x to 0.2x of the b-estradiol-induced levels, or from 0.5x to 1.0x, or 1.0x to 2.0x, or 5x to 10x. So, the authors' claim that it is "probably lower" does not seem justified; it could be lower, higher, or equal.

We have modified this part of the manuscript in light of the new results acquired.

3. Figure 5C needs better explanation in the legend. It shows 7 data circles, presumably all from PRE-containing constructs. But in Fig 5A there are only 5 PRE-containing arrangements shown. What are the other 2 data circles in Fig 5C? The 2 that don't seem represented in Fig 5A are positioned at roughly -275 and -380. This needs clarification.

We have modified panel 5A to include all the different promoter variants tested in this set of experiments. Panel 5C presents the aggregated results for all promoters, while 5B and 5D represents only a subset of the data to preserve the readability of the graphs. The figure legend has been updated to reflect these changes.

4. Page 10 and Supp Figs 7B-C: The differences in induction dynamics between pAGA1, PRE1Δ, and PRE-likeΔ look very minimal and overlapping. On what basis should we interpret these as different, rather than as essentially indistinguishable? Was a statistical test applied?

Following up on this comment, we have estimated the statistical significance of many of our results. For Panel 1E and similar graphs, we have indicated whether the output level (O) or the timing of induction (T) were significantly different than the reference curve using a t-test based on the mean of individual replicates (typically 3 or 4). For Supplementary Figure 7C, we have verified the significance of the difference between the response time of the wild type and each mutated promoter using a Wilcoxon rank sum test. For both mutated promoters, all four

replicates are significantly different from the endogenous promoter thanks to the fact that between 200 and 500 single cell measurements are compiled.

Other minor points:

5. The numbers in Fig 2E are somewhat puzzling. Fig 2D suggests that the max expression from the 23bp-TT configuration is ~ 250, compared to ~700 for the 3bp-TT configuration. This would yield ~0.35, rather than ~0.5 (in Fig 2E). Can the authors clarify the possible source of the discrepancy? Also, specifiy the induction time point used to obtain the values in Fig 2E.

Figures 2D and 2E have been updated with improved data, but the concern of the reviewer remains pertinent. In Panel 2D, we plot the median and the 25- and 75-percentile of the whole population. In panel 2E, we consider only the responding cells, based on the criteria presented in Supplementary Figure 2. This explains the different levels of expression presented in the two figures. We added a panel to display the fraction of responding cells in the WT and Ste12-EV strains in Supplementary Figure 5.

The expression output is calculated as the difference between the maximum of the nuclear relocation trace observed during the 90 minutes of imaging and the basal level prior to induction.

6. Is there a reason that the -135 construct in Figs 5A-B are not also represented in Fig 5D?

For clarity, we plotted only a limited number traces in panel 5D. and selected the most important results. Below is a figure containing the other histograms. The legend was modified to explain this better.

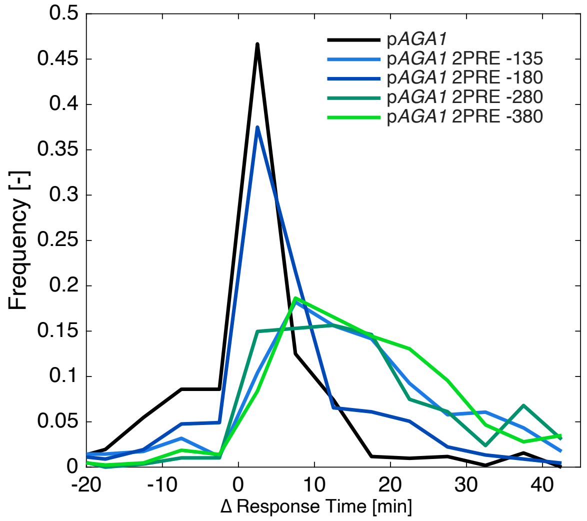

7. Page 9: "In this synthetic construct, where the nucleosome association has been inhibited...". This description is unnecessarily mysterious. It would be preferable to provide a brief description of HOW nucleosome association has been inhibited in this construct, especially because the putative nucleosomal occupancy will play an important role in later experiments.

We have used a modified *CYC1* promoter (pCYC1v3) described in Curran et al. Nat Com 2014, where the sequence of the p*CYC1* was altered to decrease the association of nucleosomes. This sequence covers mostly the core promoter and the sequence was extended by the endogenous *CYC1* sequence where the known TF binding sites were removed, along with some undesired restriction sites. We have described this synthetic construct more precisely in the manuscript and modified the sentence highlighted by the reviewer to remove this consideration about nucleosome association.

8. Page 10, line 2: I suspect this should say "-330bp" (not "-320bp").

Thank you for noticing this mistake. The value has been corrected in the revised manuscript.

9. Page 5: It would be helpful to readers if acronyms such as "pSYN_3TT" are briefly defined, to help follow subsequent parts of the text. Presumably “3TT” here means 3bp separation, tail-to-tail. If so, state that directly.

The text has been modified to define the acronym more precisely.
