# Supplementary material for: Basal association of a transcription factor favors early gene expression
Source: PLoS Genet. 2025 Jun 16;21(6):e1011710. doi: 10.1371/journal.pgen.1011710 (PMC12187013; doi:10.1371/journal.pgen.1011710)
Supplement: Attachment — Submitted filename: Letter_SPinheiro.pdf [file pgen.1011710.s011.pdf]

Dr. Serge Pelet
Department of Fundamental Microbiology
University of Lausanne

PLOS Genetics
Editorial Office

April 5, 2025

Manuscript Number: PGENETICS-D-24-00568R1

Dear Editor,

Please find enclosed our revised manuscript (PGENETICS-D-24-00568R1) entitled “Basal association of a transcription factor favors early gene expression” for publication in *PLOS Genetics*. We thank you for your time and the reviewers for their positive comments on the manuscript. We have performed the minor changes required as described in detail below.

Please do not hesitate to contact me if you need any additional information. Thank you very much for taking the time to consider our manuscript, and I am looking forward to hearing from you soon.

With best regards,

Dr. S. Pelet

*Editor Comments :*

*Dear Dr Pelet,*

*As you can see, both reviewers are happy with your revised version, and I share that opinion as well. Thus, I think the paper should be accepted. However, before I can make that decision, I have a few comments of my own that I ask you to address.*

*1- Figure 2E. Your motivating question was: is the AD of Ste12 responsible (due to its ability to dimerize/multimerize) for the ability of Ste12 to induce transcription using PREs that are "far away" (like 15bp)? In the data, the chimeras with Ste12-DBD-ERE-VP16 show only a significant (but minor) reduction in output relative to WT Ste12 for the 15 and 23 bp separation. I would conclude that the answer to the motivating question is NO, since there is plenty of output even in the absence of Ste12AD. However, in the paper it seems that it is a YES.*

From previous studies, we know that the DBD domain of Ste12 and the PRE sites on the promoter are essential for the induction of the mating genes. The question we ask is whether transcription from some of the PRE conformations are helped by the fact that the Ste12 AD domain can dimerize. For PRE spacing larger than 5 bp, the answer to this question is yes, because without this interaction (Ste12-EV) the expression output is reduced. As noted by the editor, the reduction in normalized expression output is significant but not large. The drop in expression varies from 20 to 30% for different conformations.

The text was modified to state explicitly the amount of the decrease in expression output in the Ste12-EV mutant and the conclusion was modified to explain more clearly that the self-interacting properties of the AD domain of Ste12 contribute to the overall expression output, which is mostly defined by the interaction between the DBD and the PREs.

*2- For Figure 6G (the new eviction experiment). It is not stated which primers were used in the qPCR to measure protection. Do they cover all the promoter or just the region where the PRE was placed? )(naively I thought it should be the region where the PREs were placed) Please include this information.*
*This is relevant, since the wt AGA1 promoter has the PREs in a region supposedly already mostly devoid of nucleosomes. But addition of alpha factor seems to deplete of nucleosomes anyway. Also, the way it is normalized (to the value of pAGA1 without alpha factor), suggests that we should be able to compare protection in one promoter with the others? Why is there more protection in wt pAGA1 without pheromone than in 2 PRE -330?*
*Add the statistics to compare between promoters.*

The sequence of the primers was provided in the Supplementary Data file S1. They amplify the region from -327 to -205bp. This is the same region that was analyzed by ChIP in Figures 6E and 6F, as mentioned in the figure legend and methods section. We have improved the figure legend to state this more clearly.

We designed these primer pairs to be positioned as close as possible to the PRE binding sites while maintaining consistent amplification efficiency across all synthetic AGA1 promoter variants for reliable comparison. Amplifying regions containing all PRE elements would produce large PCR amplicons (>150 bp) that exceed nucleosome size and are suboptimal for qPCR. Therefore, we strategically positioned primers near the modifications while ensuring they amplified wild-type sequence.

Regarding your observation about the wild-type AGA1 promoter, the PREs are indeed located in a region that is relatively nucleosome-poor even under basal conditions. However, this region is not completely devoid of nucleosomes. According to our previous measurements (Aymoz MSB 2018), this falls at the edge of the -2 nucleosome in the WT *AGA1* promoter. In the mutated promoters, we speculate that this -2 nucleosome can shift its central position. This could explain why for some promoter the signal in the absence of pheromone is higher or lower than in the reference p*AGA1* construct.  We have performed a statistical test and found that a significant difference between the pAGA1 and the mutated promoters is only found for the promoter with 2 PREs at -330. We have included this information in Figure 6G. However, we believe that the interesting observation is to which extent the nucleosome gets remodeled upon pheromone treatment for each promoter variant. And this remodeling is in line with the expression output measured for each promoter.

*Minor comments:*
*1- The Zenodo link does not seem to be operational.*

The Zenodo repository has now been published and is accessible.

*2- There seems to be missing statistical analysis on several Figures. For example, in all the transcription dynamics plots, such as 1D, 2C, 2D, there is no statistics to enable the reader to decide if one construct is faster than the other. Also on some of the output plots as well: no statistics on Fig3B, 4B.*

No statistical analysis has been performed on the transcription dynamics plots because they only represent one replicate and are here to illustrate the general behavior of the promoter induction. Statistically significant conclusions regarding the expression output or the timing of gene expression can be drawn from the accompanying summary graphs (1D -> 1E, 3A/3C ->3D, etc..). For the Ste12-EV experiments (2C,2D), we did not quantify the dynamics of induction because the mechanism of activation for this transcription factor is different from the endogenous Ste12. The cytoplasmic retention of Ste12-EV in the absence of estradiol prevents a fast induction of the promoter when the cells are stimulated with the hormone. As mentioned in the main text, the nuclear relocation of the constructs probably limits the speed of induction even for the endogenous pAGA1 promoter (compare Figure 2C and Supplementary Figure 2B).

We have added a statistical test to panels 3B and 4B to verify which constructs have a significantly different fraction of responding cells relative to the reference promoter.

*3- In many cases, the number of biological replicates done for each experiments is not stated. Figure 3A is just as one example. In others the reader can count the number of dots, but it would be cleaner if stated in all legends.*

To avoid making the legends unreadable, we have added the number of replicates and the number of single cells measured to the Supplementary File S1. We hope this solution is acceptable for you.

*Journal Requirements:*

*1) Thank you for stating "Raw single cell traces are available on Zenodo: 10.5281/zenodo.14438716." We couldn't access the dataset. Please provide a new link to access the dataset or provide further details to locate the data.*

The Zenodo repository has now been published and is accessible.

*2) Please ensure that the funders and grant numbers match between the Financial Disclosure field and the Funding Information tab in your submission form. Note that the funders must be provided in the same order in both places as well. Currently, "CERCA Programme of the Government of Catalonia" is missing from the Funding Information tab.*

The funding information has been updated in the online form.

*Reviewers' comments:*

*Reviewer's Responses to Questions*

*Reviewer #1: The authors have done a good job in addressing all of my comments from the previous version.*

We thank the reviewer for his favorable assessment.

*Reviewer #2: In this revised manuscript, the authors have satisfactorily addressed the issues raised in my original review. Moreover, the new data on Ste12 occupancy and nucleosome eviction in Figures 6E-6G are a very welcome addition; the results are convincing and they give substantial support to the overall story by showing that gene induction magnitude and timing are affected by features controlling the ability of Ste12 binding to compete with nucleosomal occupancy.*

*Overall, I found this to be an interesting and well-executed study. I have no serious concerns. There are a few minor points listed below that warrant correction or clarification.*

We thank the reviewer for his positive comments.

*1. Regarding Fig 4A-B, the text on page 10 states that "Extending the distance between the Ste12 binding sites and the core promoter leads to a general decline of the expression output...". Curiously, however, strong expression returns for the greatest distance (at -413), but the authors never mention this. Why is this not mentioned? And, what is the possible interpretation?*

The general trend shows a decrease of expression output as a function of increased distance, from the peak expression measured when the PRE are positioned at -223. It is true that promoters with the PRE positioned at -308 and -413 display a similar behavior, while the intermediary promoter with PRE placed at -365 is inducing at a much lower level. One potential explanation is that there is a nucleosome that is centered around -350 and limits the level of gene expression in this region. One concern with this theory is that we don't observe the additional signatures from a TF binding site that would be occluded by a nucleosome, which is a slow and noisy expression output.

The text has been modified to mention this variability in expression level as function of distance.

*2. Regarding Fig 5A-C, the numbers for the PRE positions don't match between text and Figure. The text (pg 11) says "from -128 to -435", but Fig 5A-C says "-135" and "-445". It seems these should agree with each other, no?*

Thank you for noticing this discrepancy. The difference between these numbers comes from the fact that the distance was sometimes calculated from the start or the middle of the two PRE motifs. The main text was modified to remove these differences.

*3. I think Supplementary Fig 6C should have a key to explain the symbols (of the same sort is found in Figs 3D and 4C).*

Supplementary Figure 6 has been changed to add this schematic.

*4. Minor typo: Pg 4, 9th line from bottom: "expression of" should say "expression or".*

The text has been modified.
